# Outpatient communication patterns in a cancer hospital in China: A qualitative study of doctor–patient encounters

**DOI:** 10.1111/hex.12890

**Published:** 2019-04-08

**Authors:** Jiong Tu, Ge Kang, Jiudi Zhong, Yu Cheng

**Affiliations:** ^1^ School of Sociology and Anthropology Sun Yat‐sen University Guangzhou China; ^2^ The Center for Medical Humanities Sun Yat‐sen University Guangzhou China; ^3^ Sun Yat‐sen University Cancer Center Sun Yat‐sen University Guangzhou China

**Keywords:** cancer hospital, communication patterns, doctor–patient relations, qualitative research

## Abstract

**Objective:**

The paper characterizes outpatient communication in a major cancer hospital in southern China with regard to the structure, style and focus of doctor–patient communication.

**Method:**

Fifty‐one encounters between doctors and patients were recorded in the outpatient department of the cancer hospital and analysed inductively to identify patterns of doctor–patient outpatient communication.

**Results:**

Outpatient communication in the cancer hospital is characterized by structuralized conversation, doctor domination of the conversation and a focus on technology during communication. These characteristics suggest an extreme inequality of power between Chinese doctors and patients at the individual level. They are also shaped by the institutional environment of Chinese hospitals.

**Discussion:**

Measures should be taken at both the interpersonal and institutional level to improve doctor–patient communication. At the micro‐interpersonal level, public education and professional skills training are needed to improve communication and promote mutual understanding between patients and doctors. At the macro‐institutional level, changes are needed in terms of transforming the structural factors that shape doctor–patient communication.

**Conclusions:**

Structuralized conversation, doctor domination of the conversation and a focus on technology during outpatient encounters present challenges to effective doctor–patient communication. These patterns are shaped by the institutional environment of Chinese hospitals and suggest the extreme power imbalance between Chinese doctors and patients.

## 1 INTRODUCTION

In recent years, Chinese patients have become increasingly dissatisfied with health professionals, as reflected by rising conflict and dissension.^1^, ^2^, ^3^, ^4^ Research indicates that doctor–patient disputes in China are closely related to poor doctor–patient communication.^5^, ^6^ For instance, according to a national survey, more than 70% of the medical respondents stated that inadequate communication with patients contributes to poor doctor–patient relationships.^7^ Surveys conducted with the general public and patients also reveal a close link between insufficient communication and poor doctor–patient relationships.^5^, ^8^ Worldwide, effective doctor–patient communication has been linked to patient satisfaction and trust, treatment compliance, symptom improvement and positive health outcomes,^9^, ^10^, ^11^, ^12^ while poor communication between doctors and patients is associated with patient dissatisfaction, medical mistakes and errors, disputes and malpractice litigation.^13^, ^14^ Besides, for health professionals, better communication ratings are linked to higher job satisfaction, lower feelings of stress and burnout, and fewer malpractice suits.^15^ Doctor–patient communication largely determines the doctor–patient relationship. Therefore, it is important to examine and improve doctor–patient communication in China.

Why is doctor–patient communication unsatisfactory in China? Previous researches in western societies show that doctor–patient communication is affected by multiple factors, including the patients' personal characteristics, race, gender, age, education, communication context, the physician's communicative style and situational features (eg the length of the visit, type of clinic and the physician's specialty).^16^ Besides, communication difficulty between doctors and patients is linked to the underlying differences in knowledge, culture, professional training, power and status that exist between doctors and patients.^17^, ^18^ It indicates a “clash of perspectives” between doctors who are treating diseases in clinics and patients who are coping with illness in their daily lives,^19^ as well as the mismatch between “the voice of medicine” and “the voice of the life‐world.”^20^ Yet, to what extent does Chinese doctor–patient communication differ from or remain similar to that in the western context? Previous studies in other Asian countries found under a collectivistic view, patients are conceptualized as subordinates to doctors, and the doctor is more authoritative and does much of the talking; doctor–patient communication styles tend to be more hierarchical and paternalistic in comparison to that of the United States.^21^, ^22^, ^23^ But there has been little research to characterize doctor–patient communication in China, despite a proven link between insufficient communication and poor doctor–patient relationships in Chinese hospitals. This research analyses Chinese doctor–patient communication in an outpatient department of a cancer hospital.

Communication studies are especially valuable in the case of life‐threatening illnesses, like cancer. For cancer patients, they are not only bearing the emotional burden of a cancer diagnosis, but also expected to digest complicated and often threatening information about treatment.^24^ For doctors, communication is especially challenging in the oncological setting. They face patients with varied information needs, literacy levels, and psychological conditions, and often have to break bad news.^25^, ^26^ Yet, communication between doctors and cancer patients has a powerful impact on the way in which patients make sense of, formulate decisions about, and cope with their disease.^27^, ^28^ Poor doctor–patient communication could lead to increased anxiety and uncertainty, distress and depression, non‐compliance, and coping difficulties,^25^, ^27^, ^29^ while proper communication is related to improved satisfaction and psychological adjustment, treatment compliance, and better outcome.^29^, ^30^, ^31^, ^32^ Overall, there is an urgent need to improve doctor–patient communication in cancer consultation. Doctor–patient communication in cancer consultation may be more specialized, serious, complex and frightening compared to communication in general practice.^30^ It can highlight the characters of and problems in Chinese outpatient communication. This research analyses doctor–patient communication in an outpatient department of a cancer hospital. It outlines the characteristics of Chinese outpatient communication, reveals the individual and institutional factors that contribute to poor communication and, in the process, considers measures for improvement.

## 2 METHODS

### 2.1 Data collection

The research was carried out at a major cancer hospital in a southern city in China. The hospital is one of the most renowned cancer hospitals in the country, with almost 1500 beds and over 2500 professionals. It attracts patients from nationwide, and even some from neighbouring countries. In 2017 alone, the number of outpatient and emergency visits was 922 600, and the number of inpatients was 105 500. The data for this research were mainly drawn from non‐participant observations of doctor–patient encounters in the outpatient clinics of this cancer hospital from 2014 to 2015, with some short interviews with the doctors and patients following the medical consultation, where time allowed. The method allows the engagement of multiple perspectives that is necessary in clinical communication research.

The author observed the outpatient consultations of three physicians from the Department of Thoracic Surgery: one elderly senior doctor, one middle‐aged doctor and one junior doctor. In total, seven outpatient observations were carried out. On each occasion, the researcher sat beside the doctor in the outpatient clinic and observed for a whole morning (usually from 9 am to midday). During this period, a doctor regularly saw 20‐40 patients, depending on the length of the work period. To avoid influencing the clinical encounters, the researcher did not use a recorder, but made hand‐written notes on the doctor–patient interactions and conversations while at the site, and added more details shortly afterwards. In the process, not only verbal components of the medical interview (conversations) were recorded, but also facial expressions and physical movements (interactions) between doctors and patients were written down. Ethical approval was granted by the Institutional Review Board (IRB) of the author's institution (No.: FWA00007867). Verbal consent was obtained from the physicians prior to the observation and patients (and their family caregivers involved) after the consultation. The researcher observed to saturation when all encounters began to repeat and no new information was emerging. Due to the language barrier and time limitation, the author was unable to record the Cantonese consultations or record every encounter in full. After reviewing the field notes in detail, 51 medical encounters between doctors and patients (sometimes involving the patients' relatives also) were noted down in detail. The interactions and conversations between the doctors and patients were recorded from the moment the patients entered the consultation room to the moment they left it.

### 2.2 Data analysis

This research is based on an analysis of the 51 recorded medical encounters. We conducted inductive analysis to detect the patterns of three aspects (structure, style and focus) of the observed interactions. The first and second authors coded all of the conversations and interactions line‐by‐line respectively, in the first round. They closely read all the recorded encounters and identified as many ideas and concepts as possible by attributing a conclusive sentence or words to each line (see Table 1). The medical encounters were coded with regard to the doctors' actions (eg confirming the patient's identity, medical history taking, interrupting), the patients and their relatives' behaviours (eg answering question, inquiring about alternative options, apologizing) and the conversation content (eg greetings, technology focus, social talk). All of these line‐by‐line open codes were inspected a second time by the two authors together to produce more focused coding according to the different aspects (structure, style and focus) of the communication. The focused coding in the second phase was more selective and conceptual than the initial phase line‐by‐line coding.^33^ The above three aspects were summarized in this phase by identifying the most significant or frequent codes from the first round. The authors identified that the outpatient communication was structured with an emphasis on efficiency, the style of the conversations were dominated by the doctor (through various expressions), and the focus of the conversations was hard facts (eg physical reports and test results), manifested by the prominence of technology usage and technological terms. The analysis also situates doctor–patient communication in the broad institutional setting of an outpatient encounter.

**Table 1:** Open coding example

Open coding	Conversation (in Chinese, translated by the author) D: the doctor; P: the patient; F: the patient's family member.
Confirm the patient's identity	D: Hello, is A (the patient's name) here? Who is the patient? (the patient's two family members are also at the site)
Answer	P: It's me
Medical history asking	D: Let me have a look at the physical examination results. What treatments have you got?
Answer	P: Thoracoscopy, just pulled it off
History description	F: (describes the patient's illness)
Interruption, medical history asking	D: OK (stops the description), I already know it. What was the initial symptom? (asking the patient)
Answer	P: Chest pain
Continue asking	D: Have you had a fever?
Answer	P: No
Ask for report	D: Do you have (your previous) discharge report?
	P: (looking for the report in his bag)
Ask for physical report	D: (let me) read your film
	P: (looking for the film in his bag and handing it over to the doctor)
Physical check asking	D: Have you got another film after surgery?
Answer	P: I have always taken (intravenous) drips, but no more film
Give treatment plan	D: Your illness condition requires chemotherapy, then surgery
Inquiry alternative plan	P: Is there (an alternative) biological implantation therapy?
Confirm original plan	D: No, its effect is not very good
Connect with other department	(Begins to make a call to the doctor in the chemotherapy department, a moment later)
Instruction giving	D: I just called the chemotherapy department. Go to visit Doctor X on Wednesday, or any other doctor you prefer. Do bring your pathological report then, do you understand?
Agree	P: OK
Conversation closure	D: Next (patient)

## 3 RESULTS

### 3.1 Structuralized communication with an emphasis on efficiency

**Figure 1:**
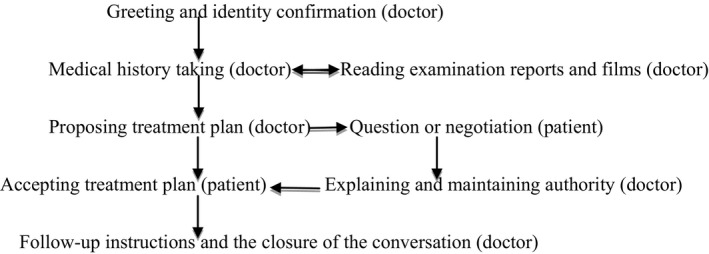
The basic structure of outpatient communication in China

As shown in Figure 1 below, a typical outpatient communication in the cancer hospital follows a standard procedure: (a) greeting and identity confirmation; (b) taking a medical history, asking about the illness condition and reading the examination report and films (occasionally, physical examination at the back of the clinic); (c) the doctor proposes a treatment plan, the patients and/or their family members question or negotiate the plan; and (d) the doctor confirms the original plan and gives further instructions, and the patients accept the plan at the end.

Outpatient interaction is a short, problem‐focused encounter. Doctors at the forefront of the cancer hospital see dozens of patients in a morning. Although the set outpatient period is from 9 am to midday, doctors frequently arrive late and leave early. Every morning, they need to make patient rounds on the wards, and then attend the outpatient clinic. Sometimes surgeries are scheduled shortly after the outpatient work. Thereby, far less time is available for outpatient consultations. Pressured by the large number of patients, doctors have only a few minutes to spend with each patient. The doctor–patient encounters in the 51 recorded cases lasted between five and ten minutes, including the time to read the reports, issue prescriptions and contact health professionals in other departments. Occasionally, the doctors spent more time with new patients who had complicated conditions. The time limitation for outpatient consultation shapes the structure of the doctor–patient communication.

The outpatient conversation tends to be brief and concise, and follows a certain pattern—the doctor asks questions and the patient answers them. The doctors need to gather information rapidly that is vital for the diagnosis and treatment. To achieve this, they must control every step of the consultation, first confirming the identity of a patient (or a patient's family member), then asking for the patient's medical history, reading the physical reports and checking the test results. After that, the doctors have normally formed a basic judgement in their mind. They then use the computer or make notes in a medical record book in order to issue a prescription. Only senior doctors who had an assistant at their side received any help with these procedures. During this phase, the doctors would briefly state their judgements and provide some instructions to the patients. In the course of an outpatient encounter, the doctors have neither the time nor the energy to pay close heed to individual patients' concerns, and rarely ask patients whether they have any questions. They do not provide much information or fully explain their diagnosis, the expected course of the illness, and the use of medication, not to mention offering health education or behavioural risk factor counselling.

On the patients' side, they need to listen closely to the doctors' questions and quickly respond to whatever they are asked. Yet, the patients and their relatives frequently provide more details than the doctors request and speak in their own terms (see Table 5) to express their concerns. Patients and their relatives who are uncertain about the illness do ask questions. As one or more relatives are often present in the clinic, the communication scenario sometimes becomes chaotic, and invariably, more than one person is heard talking at the same time. In response, doctors frequently cut off patients and their family members. Yet the “noisy” voices from the patient side may indeed remind busy doctors of something they have forgotten, as the conversation in Table 2 indicates.

**Table 2:** A typical medical interview for a new patient

(an elderly patient and his adult son entered the clinic) Doctor: What's the issue? Son: (Handing over MR film and medical report, stating the father's medical history)…… Doctor:(Looking at the film) Er, I know (from the report, you do not need to tell me) Son: (continuing reporting)…… Doctor: I already know (with rising volume, indicating to the son to stop) ……(waiting in silence) Son: (My father) had an ultrasound examination in our hometown Doctor: Oh, hand it (the report) over to me

Patients and their relatives are frequently prevented from discussing the case in detail. The doctors only wish to be told the information they need. The doctor in the conversation in Table 2 did not wish to listen to the patient's son, because he was intensely reading the physical reports and films, but the son's words did remind the doctor to look at the ultrasound report. The patients' reminders to the doctors tend to happen during the medical history taking stage. When doctors are proposing a treatment plan or giving instructions, most of the patients and their relatives would nod their heads though they have only a hazy notion of what the doctor is saying. They do not dare to challenge the doctors' professional judgement. Sometimes, patients or their relatives ask the doctors questions, which the doctors either respond to briefly, using some technical vocabulary, or ignore (eg suggesting that the staff members at the next physical check‐up or treatment stage will explain everything to them). Through this process, doctors reconfirm their authority (see Figure 1).

### 3.2 Dominant expressions by the doctors

Chinese doctors, during outpatient encounters, dominate the whole communication. They control the communication process through various styles of expression. From the 51 recorded conversations, this research identifies six kinds of expressions that Chinese doctors employ to dominate the conversation: (a) vague expressions; (b) rhetorical questions; (c) strong suggestions; (d) forbidding expressions; (e) order‐giving; and (f) interruptions (see Table 3).

**Table 3:** Examples of dominant expressions

Dominant expressions	Examples
Vague expressions	“probably” “normally” “There is a 30‐40 percent possibility that…”
Rhetorical questions	“What you want?” “What do you think?”
Strong suggestions	“You should…” “I strongly suggest that….” “I urge you to…”
Forbidding expressions	“You should never…” “You must not…” “You are forbidden from….”
Order‐giving	“Go and have a chest radiography right now!” “Go to the Nasopharynx Department”
Interruptions	“OK, I know it already” “Don't talk now” “Stop” “Next (patient)”

#### 3.2.1 Vague expressions

When doctors are questioned by patients or asked for clarification, they often use vague expressions to give a brief response. For instance, when patients request an accurate diagnosis and prognosis of their illness, doctors respond vaguely, using terms like “probably,” “normally” or “there is a 30%‐40% possibility that…” Doctors answer in various statistical or probability terms. Although the numerical information to communicate risks and prognosis seems objective, it have limitations in application to unique and individual cases.^28^ These expressions encompass many possibilities, indicating the uncertainty of cancer prognosis. They also protect doctors from giving incorrect information and prevent possible future disputes. However, these responses do not provide patients with the information they need to make a judgement and feel reassured.

#### 3.2.2 Rhetorical questions

When questioned by patients, doctors sometimes answer by asking a question in response. Rhetorical questions can be adopted during every stage of outpatient communication. For instance, when patients and/or their relatives enquire about an alternative treatment plan, the doctors may respond by asking: “What you want?” or “What do you think?” The strong tone of the doctors' rhetorical question indicates their annoyance at being questioned or challenged. The response does not provide any information, but kicks the ball back to the patients and their relatives, requiring them to take responsibility for obtaining information and making decisions if they dare to question the doctor's treatment plan. As they do not know enough about the disease, patients and their relatives do not know how to respond, and so silently accept the doctors' proposed plan.

#### 3.2.3 Strong suggestions

Strong suggestions are often made when doctors give instructions to patients. Doctors say “You should…,” “I strongly suggest that….,” “I urge you to…” to guide the patients' follow‐up treatment and health behaviours. These expressions are shaped by the doctors' professional responsibility to give suggestions to their patients and guide their behaviours. Patients normally do not feel upset by these expressions. On the contrary, strong suggestions make doctors appear responsible and conceal their continued domination of the conversation.

#### 3.2.4 Forbidding expressions

Doctors use expressions like “You should never…,” “You must not…” and “You are forbidden from….” to give instructions to their patients. The strong tone used for the forbidding expression is intended to guide patients to act in a certain way and prevent them from wrongdoing. For instance, doctors tell lung cancer patients: “Never smoke again!” The strong tone of the forbidding expression is designed to make patients realize the seriousness of the wrong act and that it is better to follow the doctors' instructions. It also has the effect of prohibiting patients from asking any questions.

#### 3.2.5 Order‐giving

Doctors also give patients direct orders to guide their next moves. These often occur during the final stage of the consultation. For instance, a doctor told a patient: “Go and have a chest radiography right now!” Order‐giving at this moment indicates the close of the conversation. Using a strong tone, the doctors indicate to the patients and their relatives, who may still be hesitating and sometimes confused, to move on quickly to the next procedure, and in the process take control of the time and speed of the communication.

#### 3.2.6 Interruptions

The above dominant expressions may still provoke questions or challenges from the patient side. On these occasions, the doctors may use interruptions to take control. Interruptions are mainly adopted by the doctors and appeared in almost every conversation recorded. Sometimes, the doctors would directly cut off the patients by saying “OK, I know that already,” “Don't talk now” and “Stop.” Sometimes, the doctors would subtly interrupt by disregarding the patients' questions, moving on to another topic, repeating or reformulating their original question, or simply calling out “next (patient)” to indicate the close of the conversation. Through determining the content of the conversation, interruptions help doctors to reinstate their agenda, make the conversation go as they want and, in the process, retain control of the speed of an outpatient consultation.

Vague expressions, rhetorical questions, strong suggestions, forbidding expressions, order‐giving and interruptions are adopted by doctors in different stages of the conversation. They aim to control the content, length and speed of communication, as in the interview extract below (see Table 4). When the patient questioned the doctor, the doctor used his “expert” claim and a raised voice to interrupt the patient, and then gave orders to the patient to guide his next move. In some of the cases observed, the doctors were angered by the patients' questions or verbal challenges, and sometimes even exploded with rage at the patients. During the outpatient encounters, the patients had to accept, listen to and collaborate with the doctors. Otherwise, conflict would ensue.

**Table 4:** Extract from a medical interview

(P: the Patient; D: the Doctor) D: From this film, I'm now sure you need treatment in the Nasopharynx department. My department is for surgery, you need chemotherapy P: What should I do next? D: Go to the Nasopharynx Department (for an outpatient consultation) P: But it's not my Nasopharynx, it's my lung (that has a problem requiring treatment) D: I know. Listen to me (raised voice), your problem is caused by a nasopharyngeal carcinoma. You need to go there (the Nasopharynx Department), and do not need to come to my Department of Thoracic Surgery P: So it was a misdiagnosis earlier? He (the previous doctor) told me come to the Department of Thoracic Surgery D: No, the previous diagnosis suggested you see an expert, now I (as the expert) am telling you…. P: What shall I do now? D: Register for (outpatient care in) the Nasopharynx Department P: There's no need to treat my lung first? D: No need

Overall, in the outpatient communication, there are two or three competing voices—the voice of the doctor, the voice of the patient and sometimes the voice of the patient's family members. An ideal conversation is based on all the participants having an equal chance to have their say. However, the above expressions are not based on the principle of equality. Doctors interrupt, order and neglect patients' voices. Their dominant expressions far outweigh the expressions of the patients, reflecting the extreme inequality of power between Chinese doctors and patients. These expressions again reinforce doctors' domination during doctor–patient conversation.

### 3.3 The focus on technology and the use of technological terms during communication

The diagnosis and treatment of cancer rely heavily on technology. Placing emphasis on technology also helps the doctors to dominate their interactions with patients. In all of the 51 conversations, the doctors asked for hard data (physical reports, test results, radiologic films, etc.) or sent the patients for high‐tech examinations. During outpatient consultations, doctors normally ask for patients' medical records and test results first. If a patient has not undergone all of the required tests, the doctors will require the patient to have a new radiologic film taken or to undergo further diagnostic tests, and then return having received the physical report. Moreover, most of the physical tests undertaken in outpatient clinics now involve a machine and the doctors rarely carry out physical checks in person, except in a few cases that this study recorded. For the doctors at this cancer hospital, the “hard” data (medical records, case reports, physical examinations and pathology tests) are reliable, objective and scientific, which enable them to see the size and nature (benign or malignant) of the tumour, while the views of the patients and their relatives are “soft” data, mainly subjective experiences and feelings, and hence unnecessary, except for some basic information.

Other reasons for Chinese doctors' overemphasis on technology include the limited time available to make a diagnosis, which means that the doctors are unable to ask for a medical history and listen to the patients in depth. The structuralized communication also encourages doctors to rely on technology, for it is direct, objective and standardized. Moreover, Chinese doctors working at the forefront of hospital assume the multi‐tasking role of an information processor, from collecting the patient's medical history, and analysing the information to taking action. They act as the gatekeepers of the hospital to decide which patients to admit and which to triage. All of these tasks must be based on objective “hard data.” The emphasis on technology use has become a common strategy for doctors to process patients quickly in a standard and objective manner. Furthermore, with the deterioration of the doctor–patient relationship in China, technological evidence now protects doctors from medical complaints and lawsuits.^34^ The over‐prescription of pharmaceuticals and high‐tech clinical tests in Chinese hospitals is also driven by the profit‐motivated behaviours of Chinese physicians, because hospitals have linked physicians' incomes to their revenue generation.^35^ In practice, by using these hard “facts,” doctors can define certain decisions as purely technical matters that do not allow the patients' to negotiate or require their consent.

Yet, doctors and patients may have a different understanding of technology use. In this study, the patients often complained that the doctors only looked at the computer or radiologic film and also noted the endless examinations and tests they had to go through. The difference between the patients and doctors' perceptions of illness and technology is also manifested in the words they used, respectively (see Table 5 below). The patients' illness narratives arise from their lived experiences, which concern the influence of their illness on their daily life. Yet, the doctors focus on scientific and accurate expressions of the disease, which involve many technical terms and much hard evidence: lesion, MRI, biopsy, thoracoscopy, ultrasound examination, etc. Although patients in general are now increasingly informative with rising internet use and some patients have high health literacy,^36^ most patients still feel confused facing complex conditions like cancer. Besides, there are many newly diagnosed patients in outpatient consultation who are unfamiliar with technical terms. Many patients feel confused about these expressions doctors use, but dare not ask many questions.

**Table 5:** Different expressions by the doctors and patients

Medical technological expressions (doctors)	Daily life expressions (patients)
A. (Pointing at the radiologic film) the lesion is here, a lymph node here, but the cut of (your) left lung biopsy is too narrow, (I) suggest you redo it B. The lesions are few, pleural dissemination, have a thoracoscopy C. Take an MRI (magnetic resonance imaging), if the problem is identified, then there is no need for a thoracoscopy D. Have a primary focus biopsy, get the cancer tissues and pathological section	A. Will I be hospitalized? Can it be fixed soon? B. During my physical examination, that doctor said there was a cyst, but that it was nothing to worry about. I'm not sure. (I) want you to have a look again C. (Pointing to his chest) It's painful here, it hurts when I cough. It started this July D. I eat far less now, and have lost more than 5 kg, but still do some farm work

On the other hand, the doctors perceive the patients' expressions to be non‐professional and subjective in nature. The doctors' emphasis on technology and the use of technological vocabulary permeate the whole process of doctor–patient communication. They enable doctors to evoke a sense of assured competence and retain control of the conversation. Yet doctor–patient communication processes include many non‐technological parts: relationship building, understanding of the patient's viewpoint and shared decision making, etc.,^37^ which are essential for a therapeutic relationship. Unfortunately, they are not a major concern for the doctors in the outpatient clinic of the cancer hospital. In the recorded conversations, social talk was generally absent, and the doctors rarely expressed empathy with or tried to comfort the patients.

## 4 DISCUSSION AND CONCLUSION

### 4.1 Discussion

The doctor–patient interaction in a hospital setting is a special interaction, with the main focus being on diagnosis and treatment. During a typical 5‐10 minute outpatient encounter, the doctors take intentional control of the structure, content and speed of the communication. The doctors need to speed up their work procedures and send patients along the line as quickly as possible to move them on to the next hospital procedure. Therefore, the communication is structuralized to follow a typical pattern with an emphasis on efficiency. Moreover, the doctors adopt various techniques (vague expressions, rhetorical questions, strong suggestions, forbidding expressions, order‐giving and interruptions) in order to dominate the conversation. The emphasis on technology is another way in which doctors retain control of the communication, as manifested by their focus on clinical data, examination reports and radiologic films. Yet, the patients and their relatives usually have additional concerns that fall outside the boundaries of the typical, narrow medical agenda, as shown by their repetitive attempts to have their say. However, patients rarely have a chance to present their views, ask questions or outline their complaints. During outpatient encounters, Chinese doctors, like doctors in many western contexts,^20^, ^38^, ^39^ play the dominant role; yet, Chinese patients have far less negotiating power in outpatient clinics compared to their peers in western societies.^3^, ^40^

**Figure 2:**
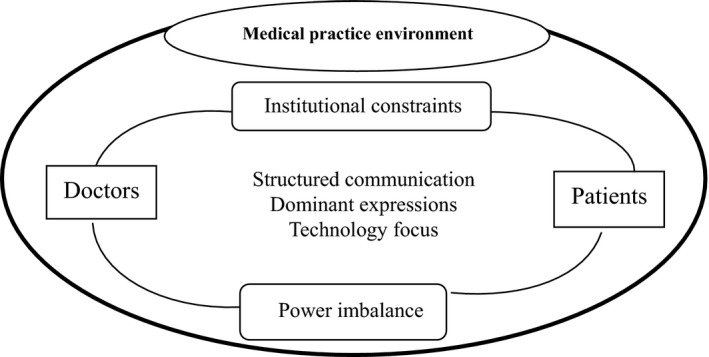
Characteristics and institutional environment of doctor–patient communication in China

Chinese doctor–patient communication needs to be situated in the practice environment in which the doctors work. Since the 1990s, most Chinese public hospitals have been required to self‐fund, with reduced state investment. In the market, public hospitals increasingly operate on a commercial basis, with an emphasis on profit and efficiency.^3^ Hospitals tie physicians' incomes to their revenue generation which undermine the quality of clinical encounters.^1^ Moreover, the medical institutions at the community level are poorly equipped. The number of patients overwhelms the upper‐level hospitals. During outpatient encounters, doctors are frequently multi‐tasking, within an extreme time limitation. They normally work alone in the outpatient clinic (except for the very senior ones, who have assistants) and have to handle all of the procedures by themselves, from medical history taking to communicating with other departments. Overburdened doctors are pressured to work with a priority on efficiency. The presence of more than one attendant for a patient further complicates this environment. One professional vs. several lay people places doctors in a chaotic setting. All of these factors force doctors to adopt a style of high control, involving structuralized, doctor‐dominated communication. The tense doctor–patient relationship further damages the communication process. In order to protect themselves, doctors tend to say less, express themselves in a professional and objective manner, and emphasize the use of technology as objective evidence to avoid litigation. Overall, the above characteristics of outpatient communication reflect the extreme inequality of power that exists between Chinese doctors and their patients. These characteristics reinforce the doctors' domination of clinical encounters. Yet, all of these factors are shaped by the Chinese health system and hospital structure (see Figure 2 below).

Effective communication is an essential aspect of quality care, especially for life‐threatening conditions like cancer. However, the above characteristics of outpatient communication in the Chinese hospital inhibit the effective mutual exchange of information that is central to good quality care. Poor communication not only makes patients anxious but also poses challenges for doctors, possibly leading to conflicts and disputes. In the cancer hospital where we conducted this research, many medical complaints are actually due to the health professionals' manners, attitudes and communication styles. Chinese doctor–patient communication needs to be improved, and measures should be taken at both the micro‐interpersonal and macro‐institutional levels.

At the micro‐level, both health professionals and patients need communication training and education. On the patient side, they should continue to be educated on how to use their time with the physicians effectively and efficiently. Public education is needed to reduce the knowledge gap between experts and lay people, and produce a mutual understanding between the patients and doctors. Meanwhile, provider education is needed on the doctor side to take greater account of the patients' views. Active patient (and family/caregiver) participation can help physicians to identify, clarify and understand the patients' goals, needs, preferences and values.^41^ Doctors should give patients opportunities to elaborate their concerns during clinical encounters, and should focus not only on information‐gathering but also information‐giving. Communication skills training may also be useful in enhancing Chinese health professionals' skills,^42^ especially those related to humanitarian care.^43^

At the macro‐level, changes may be made to remove or minimize the many barriers to effective communication in the practice environment. Many of the suggestions for improving doctor–patient communication are tailored to western contexts.^44^, ^45^, ^46^ Chinese doctors practice medicine in a different cultural, social and institutional environment.^40^ Therefore, more work is needed in terms of transforming the practice environment within which the doctor–patient encounter takes place. First, efforts should be made to reduce the institutional pressure on doctors, leaving them more time to spend with their patients. For instance, aids and assistants (eg assistant nurses, social workers) should be arranged to triage the doctors' tasks, and new technology (eg mobile applications for remote communication) can be adopted to promote communication.^47^, ^48^ Second, the marketized medical institutions should change their priority from efficiency to an emphasis on the quality of care that the patients receive. The inappropriate internal incentives that tie physicians' incomes to their revenue generation within Chinese hospitals also need to be reformed to provide more patient‐centred care.^35^ Moreover, for China's health‐care delivery system, community‐level health care should be improved to play the gatekeeper role and so reduce the pressures that doctors experience at the higher level hospitals. China's ongoing health care reforms provide an opportunity for these institutional and system changes to be carried out in the long run.

### 4.2 Conclusion

Communication between patients and doctors in an outpatient setting at a major cancer hospital in China was found to have three prominent characteristics: structured communication, doctor domination of the conversation and a focus on technology. These characteristics inhibit effective doctor–patient communication and are shaped by the respective roles of the doctors and patients, the differences in their power and knowledge, the institutional arrangements of Chinese hospitals and problems related to China's health system in general. To improve doctor–patient communication, measures should be taken at both the micro‐interpersonal and macro‐institutional levels. At the micro‐level, both health professionals and patients need communication training and education. At the macro‐level, changes are needed regarding the practice environment that creates many barriers to effective communication. Although the communication style of the doctors in different hospitals and departments varies, this study, based in one department of a cancer hospital, outlines the problems faced with regard to China's outpatient communication in general. Further studies can be conducted in many other hospitals, and experiments can be carried out to explore measures to improve communication.

## CONFLICT OF INTEREST

None.

## ETHICAL APPROVAL

Ethical approval was granted by the Institutional Review Board (IRB) of the author's institution (No.: FWA00007867).
